# *BRAF* V600E mutation in Juvenile Xanthogranuloma family neoplasms of the central nervous system (CNS-JXG): a revised diagnostic algorithm to include pediatric Erdheim-Chester disease

**DOI:** 10.1186/s40478-019-0811-6

**Published:** 2019-11-04

**Authors:** J. Picarsic, T. Pysher, H. Zhou, M. Fluchel, T. Pettit, M. Whitehead, L. F. Surrey, B. Harding, G. Goldstein, Y. Fellig, M. Weintraub, B. C. Mobley, P. M. Sharples, M. L. Sulis, E. L. Diamond, R. Jaffe, K. Shekdar, M. Santi

**Affiliations:** 1 Department of Pathology, University of Pittsburgh School of Medicine, UPMC Children’s Hospital of Pittsburgh, Pittsburgh, PA USA; 2 Department of Pathology, University of Utah, Primary Children’s Hospital, Salt Lake City, UT USA; 3 Department of Pediatric Hematology-Oncology, University of Utah, Primary Children’s Hospital, Salt Lake City, UT USA; 4 Children’s Hematology Oncology Centre, Christchurch Hospital, Christchurch, New Zealand; 5 Department of Pathology, Christchurch Hospital, Christchurch, New Zealand; 6 Department of Pathology and Laboratory Medicine, Children’s Hospital of Philadelphia, Philadelphia, PA USA; 7 Department of Pediatric Hematology-Oncology, Hadassah University Hospital, Jerusalem, Israel; 8 Department of Pathology, Hadassah Hebrew University Medical Center, Jerusalem, Israel; 9 Acquired Brain Injury Service, Alyn Pediatric and Adolescent Rehabilitation Hospital, Jerusalem, Israel; 10 Department of Pathology, Vanderbilt Hospital, Nashville, USA; 11 Department of Pediatric Neurology, Bristol Royal Hospital for Children, Bristol, England; 12 Department of Pediatrics, Memorial Sloan Kettering Cancer Center, New York City, USA; 13 Department of Neurology, Memorial Sloan Kettering Cancer Center, New York, NY USA; 14 Department of Pathology, University of Pittsburgh School of Medicine, UPMC Magee Women’s Hospital, Pittsburgh, PA USA; 15 Department of Radiology, Children’s Hospital of Philadelphia, Philadelphia, PA USA

**Keywords:** *BRAF* V600E, Juvenile Xanthogranuloma, JXG, Central nervous system, CNS, Pediatric, Erdheim-Chester disease, ECD, LCH

## Abstract

The family of juvenile xanthogranuloma family neoplasms (JXG) with ERK-pathway mutations are now classified within the “L” (Langerhans) group, which includes Langerhans cell histiocytosis (LCH) and Erdheim Chester disease (ECD). Although the *BRAF* V600E mutation constitutes the majority of molecular alterations in ECD and LCH, only three reported JXG neoplasms, all in male pediatric patients with localized central nervous system (CNS) involvement, are known to harbor the *BRAF* mutation. This retrospective case series seeks to redefine the clinicopathologic spectrum of pediatric CNS-JXG family neoplasms in the post-BRAF era, with a revised diagnostic algorithm to include pediatric ECD. Twenty-two CNS-JXG family lesions were retrieved from consult files with 64% (*n* = 14) having informative *BRAF* V600E mutational testing (molecular and/or VE1 immunohistochemistry). Of these, 71% (*n* = 10) were pediatric cases (≤18 years) and half (*n* = 5) harbored the *BRAF* V600E mutation. As compared to the *BRAF* wild-type cohort (WT), the *BRAF* V600E cohort had a similar mean age at diagnosis [*BRAF* V600E: 7 years (3–12 y), vs. WT: 7.6 years (1–18 y)] but demonstrated a stronger male/female ratio (*BRAF *V600E: 4 vs WT: 0.67), and had both more multifocal CNS disease ( *BRAF*V600E: 80% vs WT: 20%) and systemic disease (*BRAF* V600E: 40% vs WT: none). Radiographic features of CNS-JXG varied but typically included enhancing CNS mass lesion(s) with associated white matter changes in a subset of *BRAF* V600E neoplasms. After clinical-radiographic correlation, pediatric ECD was diagnosed in the *BRAF* V600E cohort. Treatment options varied, including surgical resection, chemotherapy, and targeted therapy with BRAF-inhibitor dabrafenib in one mutated case. *BRAF* V600E CNS-JXG neoplasms appear associated with male gender and aggressive disease presentation including pediatric ECD. We propose a revised diagnostic algorithm for CNS-JXG that includes an initial morphologic diagnosis with a final integrated diagnosis after clinical-radiographic and molecular correlation, in order to identify cases of pediatric ECD. Future studies with long-term follow-up are required to determine if pediatric *BRAF* V600E positive CNS-JXG neoplasms are a distinct entity in the L-group histiocytosis category or represent an expanded pediatric spectrum of ECD.

## Introduction

In the most recent revised classification of histiocytic disorders, [21], cutaneous juvenile xanthogranuloma (JXG) lesions and those JXG lesions with a systemic component, but not associated with a molecular alteration, are categorized separately into the cutaneous or “C”-group histiocytosis. However, extracutaneous JXG lesions with mitogen activated pathway kinase (MAPK) / extracellular-signal-regulated kinase (ERK) pathway activating mutations are now categorized into the Langerhans “L-group” histiocytosis, including three rare *BRAF* V600E JXG “L-group” neoplasm [56]. In this revised classification, Langerhans cell histiocytosis (LCH) and Erdheim Chester Disease (ECD), are also categorized in the “L group” of histiocytic neoplasms. At the far ends of their phenotypic spectra, LCH, ECD, and JXG all have distinct clinical and pathologic features; however, this shared categorization was proposed based on similar molecular alterations, mixed LCH/ECD histiocytic presentations in adult cases, and accumulating data supporting a common hematopoietic precursor, at least between adult LCH and ECD [21]. However, pediatric extracutaneous JXG with MAPK molecular alterations as an L-group histiocytosis, has been less studied in relation to its possible shared origins with LCH and pediatric ECD [10, 16, 38, 40, 46, 51] Furthermore, while the *BRAF* V600E mutation constitutes the majority of molecular alterations in ECD and LCH [3, 5, 30, 53], only three reported JXG neoplasms, all in male pediatric patients with localized central nervous system (CNS) involvement, are known to harbor the BRAF mutation; however, none showed evidence of systemic disease or a prior history of LCH [56].

In general, CNS-JXG neoplasm are rare, often requiring surgical resection or chemotherapy [13, 36, 55, 58] and do not have the propensity to regress spontaneously, unlike their cutaneous JXG counterpart [58]. CNS-JXG neoplasm range from isolated CNS lesions to multifocal CNS lesions to those associated with systemic disease [6, 13, 22, 26, 27, 36, 58]. In adults, CNS based neoplasms with a JXG or xanthogranuloma pathologic phenotype are often the first and most debilitating manifestation of ECD. They are often a challenge to diagnose and have a generally poor prognosis; however, in adults these neoplasms often have an excellent response to inhibitor therapy [15, 24, 48]. In children, both systemic JXG with CNS involvement and CNS-limited JXG also appear to have poorer outcomes, as compared to pediatric JXG without CNS disease; however, none of these prior pediatric JXG studies have investigated the BRAF mutational status [13, 58].

Furthermore, the current revised classification of histiocytes [21] has created a divide between the JXG family of neoplasms with molecular alterations (L-group) and those without molecular alterations (C-group). Standing alone, this grouping does not have particular clinical significance, especially given that both C-group and even R-group histiocytic lesions now also harbor MAPK-pathway activated mutations [16, 25, 28, 44, 49, 52]. Furthermore, the World Health Organization (WHO) recommends that CNS neoplasms have an initial morphologic report followed by an integrated final diagnosis after molecular studies are completed [42]. The aim of this study is to revisit the pathology and incidence of *BRAF* V600E mutations in pediatric CNS-JXG neoplasms in order to propose a revised diagnostic algorithm that requires the integration of pathology, molecular, clinical, and radiographic findings for a comprehensive final diagnosis, in the hope of advancing clinical management and treatment options.

## Materials and methods

### Cases: inclusion and exclusion criteria

Following institutional review board approval (University of Pittsburgh IRB number PRO12110055), we retrieved cases from our pathology consult files for CNS-based JXG family lesions, which includes previously published pediatric cases [14, 55, 57]. In our initial inclusion criteria, we included all cases that were diagnosed as a JXG family neoplasm by morphology and immunophenotype, as previously described [8, 50, 59]. In brief, JXG family neoplasms range from 1) small to intermediate-sized mononuclear histiocytes, to 2) abundant foamy, xanthomatous (i.e. lipidized) histiocytes and Touton giant cells, to 3) those lesions resembling benign fibrous histiocytoma with a predominance of spindle-shaped cells and fibrosis with lesser quantity of foamy histiocytes and giant cells, while also including 4) oncocytic cells with abundant glassy pink cytoplasm (i.e reticulohistiocytoma” subtype). Under the microscope JXG and ECD share similar morphologic patterns and a shared immunophenotype (i.e., positive: CD163, CD68, CD14, Factor 13a, fascin, typically S100 negative, and CD1a and Langerin negative). Both JXG and ECD can be diagnosed as “JXG family” on pathologic grounds alone, with the distinction of ECD made by correlating appropriate clinical and radiographic features, as previously described [15].

**Fig. 1:**
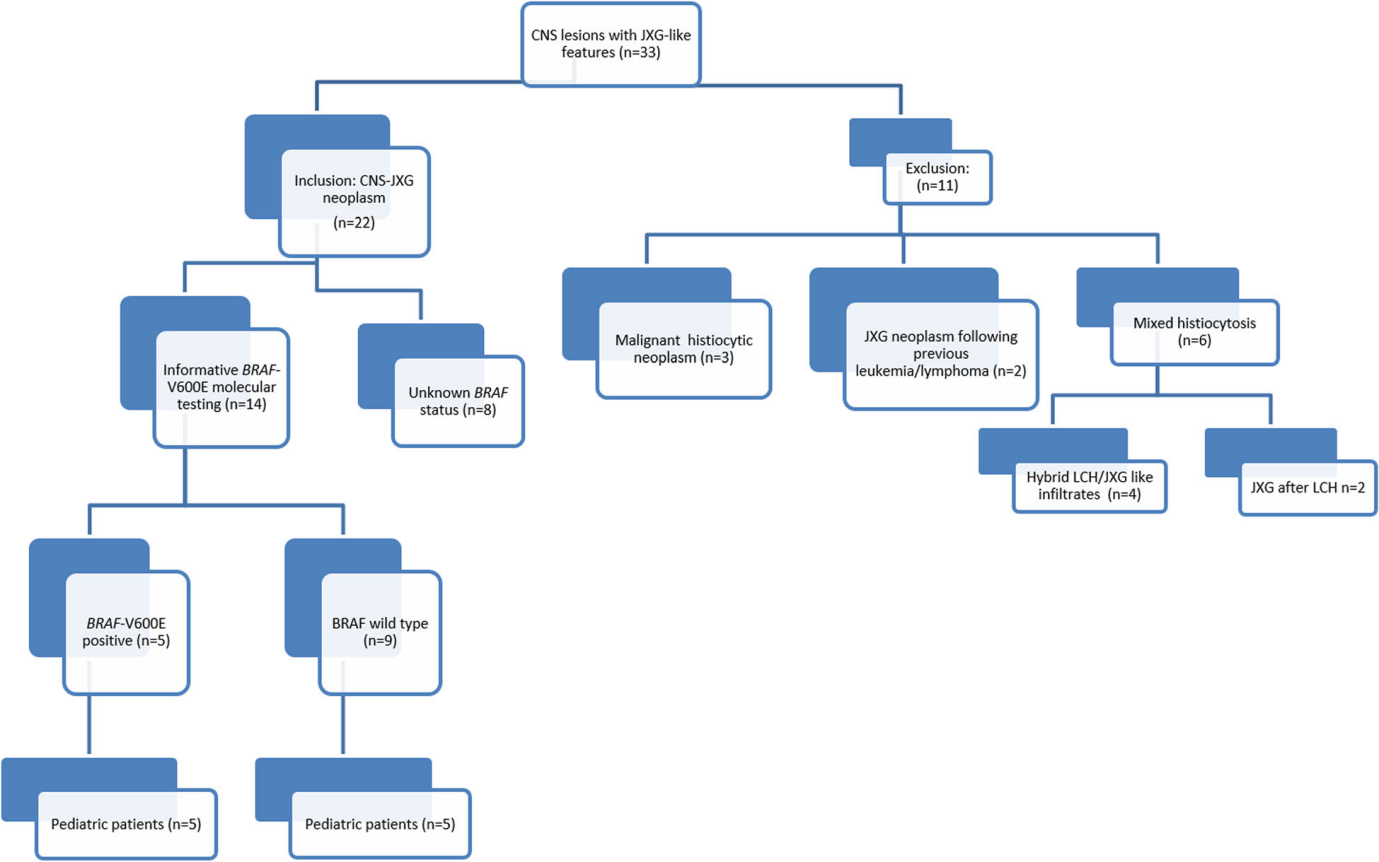
Flow Diagram of Cases. A total of 10 Pediatric CNS-JXG neoplasms with informative BRAF status are included in primary analysis

Case files were reviewed over a 20-year time span (1998–2018). Detailed clinical, radiographic, and therapy-related data were collected for all available patients. Exclusion criteria comprised those CNS-JXG cases with a mixed histiocytic phenotype (*n* = 6), including LCH either concurrently or previous to the CNS-JXG diagnosis; CNS histiocytic sarcoma with JXG immunophenotype (*n* = 3), and CNS-JXG following leukemia (*n* = 2) (Fig. 1), as these lesions carry different biologic potentials.

### Immunohistochemistry

Immunohistochemistry was performed on 3 μm thick formalin fixed paraffin embed (FFPE) sections using commercially available antibodies: CD163, CD68 PGM1, CD14, Factor XIIIa, Fascin, Ki-67, S100, CD1a, Langerin, and Braf-VE1 (Table 1).

**Table 1:** Immunohistochemistry of histiocytic neoplasms

Antibody (Source)	Clone	Dilution	Antigen Retrieval (Ventana proprietary reagents)	Detection (Ventana proprietary reagents)
CD163 (Novocastra)	10D6	1:200	uCC1 mild	iView DAB
CD68 (Dako)	PG-M1	1:100	uCC1 mild	iView DAB
CD14 (Cell Marque)	EPR3653	1:100	uCC1 standard	Optiview DAB
Factor XIIIα (GeneTex)	Polyclonal	1:250	Protease	iView DAB
Fascin (Dako)	55 K-2	1:500	uCC1 mild	iView DAB
Ki-67 (Dako)	MIB-1	1:25	uCC1 mild	iView DAB
S100 (Dako)	Polyclonal	1:3000	uCC1 mild	iView DAB
CD1α (Immunotech)	O1O	1:5	uCC1 mild	uV DAB
Langerin (Leica)	12D6	1:100	uCC1 standard	uV DAB
BRAF V600E (Ventana Medical Systems)	VE1	Pre-dilute	uCC1 standard	OptiView DAB

### BRAF V600E assessment

*BRAF* status was assessed either by DNA-based studies and/or immunohistochemistry with a clinically validated BRAF V600E (VE1) immunohistochemical stain (Table 1). Previous in-house validation and others have shown a very high correlation with molecular status when diffuse 2–3+ intensity granular staining is present in > 10% of while negative cases had either complete absence of staining, weak/faint granular staining (1+) or staining in only rare, single scattered cells, often not possessing the morphology of a histiocytic cell [41]. For those that had molecular testing around the time of diagnosis, a variety of methodologies were used, dependent on the referring institution. A single sample (case 3) underwent PCR Sanger Sequence locally at our institution on available FFPE consult block material of the CNS-JXG lesion, along with Braf-VE1 immunostaining. Briefly, a manual microdissection was performed on this sample (> 50% tumor cells present). DNA was isolated using standard laboratory procedure with optical density readings. For the detection of the mutation, Light Cycler Platform (Roche Molecular Systems, Inc. Pleasanton, California) was used to amplify BRAF exon 15 codons 599–601 sequences. Post-PCR melt curve analysis was used to detect mutation and confirmed with Sanger Sequencing of the PCR product on ABI3130 (Applied Biosystems, Thermo Fisher Scientific, Waltham, Massachusetts). The limit of detection was approximately 10–20% of alleles with mutation present in background of normal DNA.

## Results

**Fig. 2:**
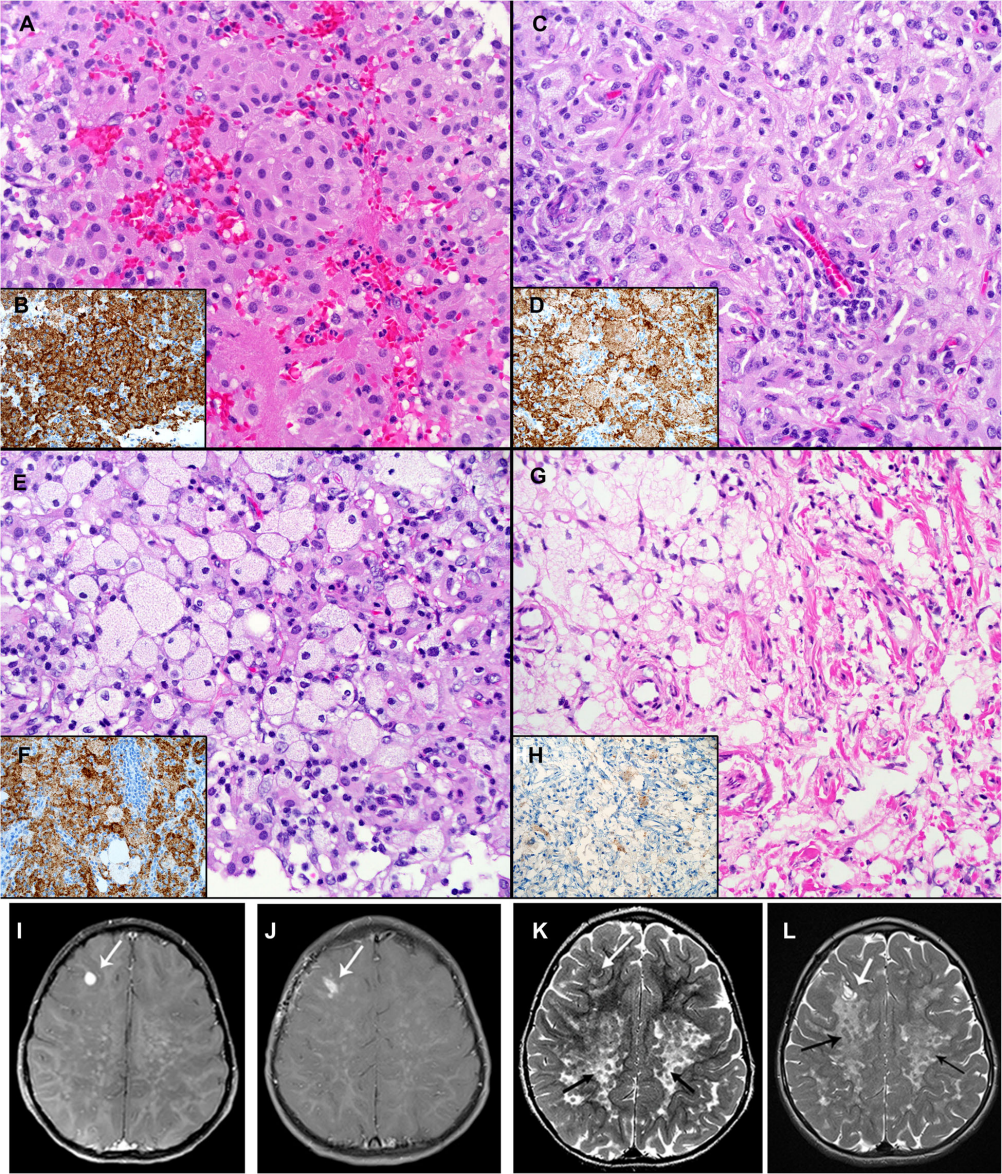
Morphologic, BRAF-VE1 expression, and CNS radiographic features of *BRAF* V600E CNS-JXG neoplasm. Various histiologic patterns in one lesion including: **a** Epithelioid histiocytes (**h**&**e**) with **b** strong (3+) diffuse BRAF-VE1 staining of histiocytes **c** Plump, pale histiocyes with **d** moderate (2+) diffuse BRAF-VE1 staining including some foamy histiocytes. **e** More abundant foamy/xanthomatous histiocytes with **f** variable moderate (2+) to weak (1+) to focally BRAF-VE1 negative xanthomatous histiocytes, and **g** fibrohistiocytic areas with only weak (1+) BRAF-VE1 staining in focal histioyctes with others negative. Original magnifications at 400x. **i**-**l**. MRI imaging showing **i** T1 axial with contrast pre-biopsy with dominant focal enhancing lesion in the right frontal lobe (white arrow) and **j** status post-excisional biopsy. **k** T2 axial with extensive confluent, nearly symmetric white matter T2 hyper-intensity throughout the cerebral hemispheres, with a posterior predominance, and a mottled appearance (black arrows) and dominant right frontal lobe lesion (white arrow), **l** status post excisional biopsy with a small amount of CSF fluid in the surgical bed and peripheral enhancement along the surgical tract (white arrow) with innumerable nodular mottled T2 hypo-intensities throughout a background of diffusely abnormal hyper-intense T2 white matter abnormalities in the bilateral cerebral hemispheres (black arrows)

We identified 22 CNS lesions with JXG phenotype that met our initial inclusion criteria (Fig. 1), designated as CNS-JXG. Fourteen CNS-JXG cases (64%) had informative molecular status for the *BRAF* V600E point mutation, which included 10 pediatric CNS-JXG neoplasms that were included in the primary analysis. The overall mean age was 7.3 years (range: 1–18 y) with a male/female ratio of 1.5 (Table 2). Within such a small cohort it is difficult to ascertain clinically relevant, statistical difference between the pediatric *BRAF* V600E (*n* = 5) and BRAF wild-type (n = 5) CNS-JXG cohorts, but certain trends were noted. There was a similar mean age in both cohorts [*BRAF* V600E: 7 years (3–12 y) vs *BRAF* wild-type 7.6 years (1–18 years)]. While the overall male/female ratio in the pediatric CNS-JXG cohort was male predominant (1.5), the *BRAF* V600E cohort had more males (male/female ratio: 4.0), as compared to the wild type cohort (male/female ratio: 0.67). The pediatric *BRAF* V600E cohort also had more cases of multifocal CNS disease [*BRAF* V600E: 3/5 (60%) vs the *BRAF* wild-type: 1/5 (20%)] along with associated CNS white matter changes and enhancement of nodular lesions (Table 2). The two cases with systemic disease were *BRAF* V600E positive (Table 2). One had multifocal CNS-JXG disease, including intracranial, sellar, dural, ventricular, and cavernous sinus involvement, along with bilateral long bone sclerosis and confirmatory bone biopsy also with the *BRAF* V600E mutation (case 3). Thus the integrated final diagnosis with pathology and radiographic correlation was that of pediatric ECD, as previously published [14]. The second case also had systemic disease, with an associated cutaneous *BRAF* V600E positive JXG lesions. Symmetric CNS white matter changes were present on MRI (Fig. 2i-l), along with an enhancing parenchymal mass; however, there was no evidence of bone involvement or other classic features of ECD. One of the *BRAF* V600E cases with multifocal CNS lesions had visual decline and panhypopituitarism from sellar/optic chiasm-based masses, while the other had resultant encephalomalacia and brain atrophy with progressive developmental delay and was started on hospice care six years after initial presentation (Table 2). In contrast, the BRAF wild-type cohort had more isolated CNS lesions without mention of associated symmetric white matter changes or reported systemic disease; however, one of these cases did not have long term follow-up after diagnosis (Table 2). The BRAF wild type group also did not have further molecular testing or phosphorylated-ERK staining.

**Table 2:** Pediatric CNS-JXG cases with *BRAF* V600E status with clinical, imaging, and treatment findings

Case	BRAF V600E Status	Sex	Age at diagnosis (y)	Radiology/ Location	Systemic Disease	Treatment and follow-up
Pediatric *BRAF*-V600E CNS-JXG patients (*n* = 5).						
1	Mutated	M	3	Unifocal: Pituitary stalk thickening and loss of bright spot on MRI.	No; LCH staging did not reveal additional lesions, including PET and CT of chest and abdomen	Presumptive MRI diagnosis of LCH with DI, started on LCH III based Rx (Prednisone and vinblastine) with mild progression of the lesion and suprasellar extension at 12 weeks. Bx performed with Rx continued for 12 mo. Stable thickening of pituitary at 2.5 y following Rx completion. No other lesions noted with central diabetes insipidus.
2	Mutated	F	4	Multifocal: Enhancing, dominant, right frontal lobe lesion, along with T2 hypointense cerebral, cerebellar, and brain stem nodules with background of symmetric T2 white matter hyperintensity.	Yes-systemic with JXG BRAF-V600E positive cutaneous lesions; but: no imaging suggestive of classic ECD lesions	Clofarabine and dexamethasone (cycle 4, 4 months after initial biopsy), demonstrating clinical and radiographic improvement
3*	Mutated	M	7	Multifocal: Enhancing intracranial, dural plaque-like thickening, sellar/suprasellar, 4th ventricle, posterior fossa, cavernous sinus	Yes –ECD confirmed on bone scan and bx with bilateral tibial sclerosing lesions	Partial resection/debulking with progression after 6 mo. 2 cycles clofarabine with progression. After dx of ECD, started on Anakinra 2 mg/kg. At 2 y f/u MRI improvement of CNS and osseous lesions.
4	Mutated	M	9	Multifocal: Enhancing Intraventricular and subependymal masses (bilateral), enlarged pituitary, Increased T2 signal pons, WM of cerebellum including dentate nuclei, post-enhanced T1 signal increase of basal ganglia (bilateral), expansion of cavernous sinuses (bilateral) (Ddx included LCH)	N – Multiple body imaging including MRI of spine, negative skeletal survey	Progressive ataxia since 4.5 yo. Imaging 4 mo after bx with extensive enhancing lesions suggestive of ‘perivascular spread’ abnormal WM signal (FLAIR): bihemispheric subcortical areas (subinsular, thalami, basal ganglia), WM of temporal/parietal/occipital lobe, and WM of cerebellar hemispheres, brain stem, mesial temporal (Comment concerning for malignant histiocytosis or demyelinating disease). 23 mo after bx: Encephalomalacia, atrophic changes of the brain, bright T2 both in WM of cerebellum, no enhancing lesions 4 y after bx: Developmental delay with ventriculomegaly and periventricular WM T2 prolongation; Persistent, stable cerebellar WM T1 and T2 prolongation without enhancing lesions 5 yr after dx: Lost to f/u in hospice care
5	Mutated	M	12	Multifocal: Large sellar mass (pituitary and optic chiasm), (enhancing) two dural based temporal masses, two lateral intraventricular masses and cerebellar ND-white changes.	No; After tissue diagnosis: no imaging s/o of ECD lesions. Subsequent MRI showed ND changes in cerebellum	DI since age 7yo, then 4th cranial nerve palsy, decreased visual acuity. MRI and Bx (originally called RDD) with subsequent resection of temporal mass; following 9 mo later with surgical decompression of optic chiasm and panhypopituitarism with cognitive decline. Started on dabrafenib (100 mg ×2/day) with dramatic clinical response. MRI 2 years on I-Rx showed no new lesions and moderate reduction tumor size. No other new lesions noted; with occasional CSF drainage through Ommaya reservoir for intermittent headache.
Pediatric BRAF wild type CNS-JXG patients (*n* = 5).						
6**	Wild-type	M	1	Unifocal: Cerebellopontine Angle with encasement of cranial nerves V and VI; MRI heterogenous T1 iso to hypointense and T2 hypointense with contrast enhancement	No	Initial radiographic concern for malignant ependymoma. Gross total resection. Started on prednisone/vinblastine. Postoperative MRI about 1 year with no evidence of recurrent disease. Unchanged postsurgical appearance of the brain including linear/nodular enhancement along the medial aspect of the left posterior fossa resection cavity that is favored to represent postsurgical change.
7	Wild-type	F	2	Unifocal Thalamus	No	Right hemiplegia at presentation that persists. IV methylprednisolone, follow-ed by oral Prednisolone at presentation that was weaned. No subsequent treatment. Followed with q6 months and now annual MRI brain surveillance scanning with no significant change in the size of the lesion and no new lesions in brain or elsewhere at 4 y follow-up.
8^	Wild-type	F	3	Multifocal: Cranial nerve/trigeminal involvement with Meckel’s cave, cavernous sinus, orbit, dural, skull base extension	No	Subtotal resection with progression and leptomeningeal spread at 9 mo and 12 mo f/u, managed with prednisone/vinblastine and then cladribine. 18 mo after chemotherapy and 3 yr after surgery, clinically stable with marked shrinkage of multifocal tumor without adjuvant therapy
9	Wild-type	M	14	Unifocal: Intraparenchymal right temporal lobe	No	Presentation with new onset seizures, hyponatremia, F/u: NA
10	Wild-type	F	18	Unifocal: Enhancing (homogenously) dural based lesions within the right frontal region (4.4 cm) with regional mass effect and vasogenic edema without restricted diffusion	NA	No significant prior medical history presents with 2 wk. history of progressive worsening bifrontal headaches, dizziness, and intermittent left periorbital and hand paresthesia. After resection, chronic, recurrent headache. 2 year post-resection MRI head surveillance imaging no recurrent tumor

Legend: *M* Male, *F* Female, *y* year, *yo* years old, *mo* months, *bid* bis in die (twice a day), *ECD* Erdheim Chester Disease ,*LCH* Langerhans cell histiocytosis, *DI* diabetes insipidus, *bx* biopsy, *dx* diagnosis, *bx* biopsy, *s/o* suggestive of, *f/u* follow-up, *NA* not available, *Rx* treatment, *ND* neurodegenerative, *WM* white matter

*Consult cases with subsequent published results: *Ref 16 (Diamond et al. *Blood* 2016). ^Ref 55 (Tamir et al. *J Clin Neurosci* 2013). **Ref 57 (Tittman et al. *Otolaryngology Case Reports* 2019)

## Pathologic features of the pediatric BRAF informative cohort

The presence of the *BRAF* V600E mutation did not appear to confer a selective morphologic pattern (Table 3). Both cohorts displayed varied histologic features within the morphologic (Fig. 2) and immunophenotypic spectrum of JXG-family (Table 3). Nine of the pediatric cases had S100 available for evaluation. Two *BRAF* V600E cases and three wild-type cases had scattered S100 positive Rosai- Dorfman-Destombes Disease (RDD)-like cells, along with an additional case in wild-type cohort with multinucleated giant cells and rare cells with emperipolesis, despite no S100 expression (Table 3). Half of the pediatric cases had assessment by Ki-67/MIB-1 immunohistochemistry, with an overall low proliferation index (0–15%) when accounting for intermixed inflammatory cells. The two *BRAF* V600E cases had subjectively lower median proliferation rate (2%), as compared to the three pediatric wild-type cases (15%) (Table 3); however, there are too few cases to draw statistical conclusions on these results. Focal mild cellular pleomorphism was noted in both groups, but there was no evidence of frank anaplasia or diffuse atypia. Only one of the BRAF wild-type cases (case 6) had central ischemic type necrosis (Table 3). The BRAF-VE1 immunostain showed diffuse, strong (2–3+) granular cytoplasmic expression in the majority of the lesional histocytes (> 75%). However, there was variable staining expression noted in the different JXG-histiocyte subtypes, including within a single lesion. For example, diffuse strong (3+) VE1 expression was noted in epithelioid and finely vacuolated JXG cells, diffuse but moderate (2+) expression in foamy/xanthomatous JXG cells, and weak to negative staining (0–1+) in the fibrohistiocytic JXG component that had more heavily xanthomatous/lipidized cells intermixed with fibrosis/gliosis (Fig. 2). All wild-type cases had a mixture of cell types, with negative staining in the epithelioid/finely vacuolated and foamy/xanthomatous JXG cells (Table 3).

**Table 3:** CNS-JXG cases with *BRAF* V600E status and pathologic correlation

Case	*BRAF* V600E Status	BRAF DNA	BRAFVE1 IHC	Morphology	CD163	CD68	CD14	Factor XIIIa	Fascin	S100	CD1a	Ki-67
1	Mutated	POS	POS	Xanthomatous	na	Y	na	na	na	na	N	na
2	Mutated	POS	POS	Foamy/finely vacuolated to epithelioid, mild atypia, Touton-like GC, glial/fibrosis	Y	Y	Y	Y	Y	N	N	3%
3*	Mutated	POS	POS	Foamy/finely vacuolated with intermixed xanthomatous and Touton GC	Y	Y	Y	Y	Y	N	N	1%
4	Mutated	NA	POS	Foamy/finely vacuolated with xanthomatous, and intermixed glial/fibrosis	na	Y	Y	Y (variable)	Y	Y (light, few RDD like cells w emperipolesis	N	na
5	Mutated	POS	POS	Foamy/finely vacuolated, with xanthomatous, and intermixed glial/fibrosis	Y	na	Y	Y	Y (light)	Y (very focal and light, focal cells w emperipolesis	N	na
6**	Wild-type	NEG	NEG	Monomorphic small epithelioid cell with MNGC but not Touton type, mild pleomorphism, focal central necrosis, increased mitoses	Y	Y	Y	Y	Y	N	N	15%
7	Wild-type	NA	NEG	Monomorphic small epithelioid cell to finely vacuolated individual cell necrosis	Y	Y	Y	Y (focal)	Y (focal)	Y	N (rare small cell pos)	15%
8^	Wild-type	NA	NEG	Foamy/finely vacuolated, spindled, glial/fibrosis, rare Touton GC and rare emperipolesis in non-RDD like cells (cauterized) growth around Nerve trunks	Y	Y	Y	Y (light)	Y	N	N	5–10%
9	Wild-type	NA	NEG	Plump/finely vacuolated, with fibrous bands, and high inflammation with mild pleomorphism and Touton GC emperipolesis	Y	Y	Y	Y (light in RDD, dark intermixed)	Y	Y (light in RDD like cells)	N	na
10	Wild-type	NA	NEG	Focally xanthomatous (dural), but mostly monotonous intermediate sized finely vacuolated to epithelioid Touton-like GCs, rare emperipolesis but in non-RDD cell, admixed lymphocytic inflammation	Y	Y	na	Y	na	N	N	na

Legend: Y = yes expression of listed immunostain; N = no expression; NA-not available, MNGC = multinucleated giant cells; RDD-Rosai-Dorfman Destombes Disease (Definition of RDD-like cells: cell with either emperipolesis and/or S100 expression but do not confirm in totality to RDD, either by non-diagnostic cytomorphology features or only single scattered cells without cluster/aggregates)

*Consult cases with subsequent published results: *Ref 16 (Diamond et al. *Blood* 2016). ^Ref 55 (Tamir et al. *J Clin Neurosci* 2013). **Ref 57 (Tittman et al. *Otolaryngology Case Reports* 2019)

## Therapy and outcomes of the pediatric BRAF informative cohort

Treatment options in pediatric CNS-JXG cases were variable showing a combination of both surgical excision and systemic chemotherapy (Table 2). For most *BRAF* V600E CNS-JXG cases, the *BRAF* mutational status was not known at the time of initial diagnosis. Treatments included the following: LCH III based protocol with prednisone/vinblastine for 12 months in unifocal CNS disease of the hypothalamic-pituitary axis, clofarabine and dexamethasone for systemic JXG with multifocal CNS-JXG, anakinra for pediatric ECD that had previously progressed on prednisone/vinblastine for 6 weeks, cladribine for 6 cycles, and clofarabine for 2 cycles [14], and BRAF-inhibitor therapy with dabrafenib for a multifocal CNS disease, which was aggressive and refractory to first line therapy. In this last case, dabrafenib showed an immediate and dramatic clinical response, including complete resolution of hyperventilation and weaning from dexamethasone with interval MRI at 2 months, 4 months and 15 month of therapy, along with a reduction in intracranial size and no new lesions. (Table 2). Case 4 did not have a known *BRAF* V600E prospectively in his course with progressive CNS white matter disease in the years following excision. The wild-type cases also had surgical resection with initial prednisone/vinblastine and then cladribine in one case with multifocal lesions and prednisone/vinblastine in a unifocal lesion of the cerebellopontine angle of a 1-year-old (Table 2).

## Discussion

This retrospective case series characterizes the largest series to date of *BRAF* V600E mutated pediatric JXG family neoplasms, all of which were first diagnosed with CNS disease and share a striking young male predominance with aggressive disease. As compared to the three previous reported *BRAF* mutated cases [56] and our *BRAF*-wild type case, there was a similar age distribution throughout, but overall more boys are represented in the *BRAF* V600E cohort. Radiographically, the majority of *BRAF* V600E CNS-JXG neoplasms had multifocal CNS disease, often with contrast enhancement and a subset were noted to have background white matter changes, suggestive of neurodegeneration, which is also a feature shared in cases of CNS-ECD and CNS-LCH [15, 45]. Two of our *BRAF* V600E CNS-JXG cases also presented with systemic disease, including one classic pediatric ECD with long bone involvement and one case with cutaneous JXG and associated CNS white matter disease. In both cases, the non-CNS systemic lesions also demonstrated the *BRAF* V600E mutation. Treatment options varied in this case series, but those with *BRAF* V600E may benefit from targeted inhibitor therapy, especially in aggressive or refractory disease and may halt progressive decline from histiocytosis-associated neurodegeneration, which is now recognized as a *BRAF* V600E driven progress [32, 43, 45]. Together with previous published cases [56], our findings support the classification of CNS-JXG neoplasms with *BRAF* V600E into the current “L group” histiocytic neoplasm category [21], with all CNS-JXG neoplasms benefiting from upfront molecular testing including MAPK/ERK pathway mutations and possibly also *ALK* fusions/mutations [12]. Thus for clinical-pathologic relevance in CNS lesions, we propose that the neuropathologist first focus on an accurate diagnosis of CNS-JXG neoplasm. Recognizing the varied histologic subtypes and shared immunophenotype with ECD is foremost. Following this, integration with molecular testing and clinical/radiographic staging will allow for a more comprehensive, integrated final diagnosis, similar to the current WHO process for other CNS neoplasms. Furthermore, recognizing malignant cytology [47] or a previous diagnosis of leukemia/lymphoma in the same patient [9], or an associated histiocytosis including LCH [38] (either concurrently with the CNS-JXG neoplasm or previously diagnosed in the same patient) is also imperative, as all three of these instances will have different and distinct outcomes. This study specifically excluded such cases, including mixed histiocytosis, which needs further investigation to understand whether *BRAF* V600E mixed pediatric CNS LCH-JXG lesions also share a common hematopoietic precursor, similar to adult *BRAF* V600E LCH-ECD histiocytosis [4, 34]. Thus, by encompassing a comprehensive diagnostic algorithm for CNS-JXG neoplasms with morphology, molecular, clinical, and radiographic correlation, the neuropathologist will enable a heightened awareness amongst the clinical team for appropriate management and treatment, including prevention of *BRAF* V600E driven neurodegeneration, similar to LCH [45].

The pediatric *BRAF* V600E CNS-JXG neoplasms in this series share histologic and variable clinical/radiographic overlap with adult ECD cases, including one classic pediatric ECD. The other *BRAF* mutant cases, including the systemic cutaneous case with CNS-white matter changes, are suggestion of pediatric ECD, despite no diagnostic long bone involvement or other classic radiographic ECD findings, as described in adults [15]. In fact, pediatric ECD may present differently than adults and often experience a delay in diagnosis from months to years, given the rare reporting in the literature [37–39]. Since there are so few pediatric examples, it may be difficult to know the full clinical-radiographic spectrum of pediatric ECD, which in part may be due to underreporting in the pre-BRAF era. While in an adult, a nodular parenchymal *BRAF* V600E CNS-JXG diagnosed neoplasm with background CNS white matter changes and a cutaneous *BRAF* mutated xanthogranuloma lesion is highly suggestive of ECD [23], in children this presentation is not as well recognized as a form of pediatric ECD, especially in the pre-BRAF era [7]. In children, isolated skin JXG lesion are not known to harbor the *BRAF* V600E mutation (i.e., grouped as “C group” lesions) [49, 56]; however, in adults a cutaneous *BRAF* V600E xanthogranuloma is highly correlative with ECD, especially xanthelasmas, and should immediately prompt further clinico-radiographic investigation for ECD after biopsy diagnosis [15]. Thus, we propose that the same should be true for pediatric CNS-JXG lesions in which a morphologic diagnosis is only the first step in diagnosis. While our CNS-JXG pediatric patient, with an associated *BRAF* V600E skin lesion did not have classic radiographic stigmata of ECD and has thus far responded to clofarabine and dexamethasone with clinical and radiographic improvement, the background radiographic features suggestive of ECD-related neurodegeneration should be further followed in this setting. Furthermore, two other *BRAF* V600E positive CNS-JXG cases in our series also had features suggestive of ECD with progressive multifocal CNS disease resulting in cognitive decline, including brain atrophy. Despite the lack of long bone sclerosis or other classic ‘adult-type’ ECD findings, our cases not only share similarities with the aggressive cognitive decline that are observed in adult ECD, but also share radiographic features including associated white matter changes and brain atrophy [15, 18, 23, 29, 45].

For these reasons, adult ECD cases with CNS involvement are generally associated with poor prognosis [2]. Similarly, in one of the largest studies of previously published CNS-JXG [58], there was a higher rate (18.6%) of mortality/morbidity in both the isolated CNS-JXG neoplasms and those associated with systemic disease, as compared to the low mortality/morbidity (1–2%) of JXG in general [13, 36]. However, none of these previous JXG studies or registries included molecular testing, which would likely help further stratify patients, given our emerging data. In fact, one aggressive multifocal *BRAF* V600E CNS-JXG in this series, originally diagnosed in the pre-BRAF era, had poor prognosis with a rapidly progressive CNS disease with transition to hospice care, while the other *BRAF* V600E case, diagnosed prospectively, benefited from initiation with upfront BRAF-inhibitor therapy and had a dramatic and quick clinical response.

This type of immediate and favorable response is similar to the BRAF- and MAPK-inhibitor therapy in both adult ECD and LCH patients [17, 19, 24, 31]. However, this study was not designed to assess what constitutes best treatment protocols. It rather only highlights the lack of standard treatment protocols among the various cases. Treatment for CNS-JXG lesions should first take into account the final integrated diagnosis based on accurate morphologic diagnosis with molecular correlation and clinical/radiographic staging. However, in order to draw meaningful conclusions and develop consensus guidelines, long-term systematic study of these rare patients with follow-up is needed. To this end the Histiocyte Society’s International Rare Histiocytic Disorders Registry (NCT02285582) and subsequent prospective studies are poised to help accomplish this endeavor.

In the post-BRAF era, we now turn our attention to the molecular classification of histiocytic neoplasms as an area of ongoing, active investigation, which now includes extracutaneous JXG with *BRAF* V600E and MAPK pathway mutations, in addition to LCH and ECD with *BRAF* V600E mutations, and even rare reports of RDD with *BRAF* V600E [25, 44]. Thus, the question of whether the L-group histiocytic group should include only LCH/ECD or whether a more inclusive category of “MAPK-pathway activated histiocytoses” should now exist for all groups will need further discussion. Nonetheless, histology remains a seminal discriminator, as many other CNS tumors carry the *BRAF* V600E mutation, including both primary CNS (i.e., pleomorphic xanthoastrocytoma, ganglioglioma, pilocytic astrocytoma, papillary craniopharyngioma) and metastatic CNS tumors (i.e., melanoma, carcinomas including colorectal cancer). Thus, it is of foremost importance that the pathologist accurately diagnose these histiocytic neoplasms, with heightened awareness of their varied histopathologic patterns within the rubric of JXG family neoplasms, which may include ECD [8, 59, 60]. The radiologist must also be aware of their varied radiographic presentations as focal, multifocal, and possible association with white matter changes and brain atrophy, which can further progress years after surgical excision of the main enhancing parenchymal lesion. We advocate applying a consistent JXG-immunostain panel, including molecular based immunostains that will aid in the pathologic diagnosis of these neoplasms, given their variable morphologic features. It is also important to exclude other histiocytosis, including LCH both by morphology and CD1a/Langerin immunostains and RDD by morphology of large RDD histiocytes (with and without emperipolesis) with diffuse, dark S100/fascin immunostains [50]. At least one case in our series carried an erroneous diagnosis of RDD based on a subset of scattered S100 positive cells. Typically S100 immunostain has limited value in the CNS lesions with high background staining; however, a subset of CNS-JXG cases in this series had variable light nuclear and cytoplasmic S100 staining in the lesional histiocytes, with and without emperipolesis. This light staining pattern with S100 in a subset of JXG cells should be distinguished from CNS-RDD, which has strong/diffuse S100 and fascin staining of lesional histiocytes and lacks Factor XIIIa staining. Scattered RDD-like cells with emperipolesis and variable light S100 staining has been previously noted in cutaneous JXG-family lesions [33, 54]. Furthermore *BRAF* V600E mutations have also been identified in rare cases of RDD [25, 44], including a variant BRAF mutation with CNS disease [52], which further emphasizes that morphology combined with molecular are useful for accurate diagnosis.

A significant limitation of our study is the retrospective nature of this case series with limited follow-up and inability to test the BRAF-wild type cohort for additional MAPK pathway mutations. An immunohistochemical stain for phosphorylated ERK (p-ERK) is commercially available which can provide additional evidence for upregulation of the MAPK pathway as evidenced by diffuse expression in the majority of histiocytes [11, 35]. Unfortunately, many cases had no additional material to perform pERK staining. As advocated in other histiocytoses, especially those that fail standard therapy [1], the finding of MEK-ERK pathway mutations and/or upregulation by pERK may allow for more directed, targeted therapy with improved outcomes. While targeted therapy is not necessarily curative in most cases [20], it does provide a rapid and sustaining clinical response across the “L” group histiocytosis [16, 19, 32] in which there is immediate clinical response. In addition, it has value in CNS based disease not amenable to complete resection and/or in those cases that do not respond to traditional therapy protocols, including histiocytosis- associated neurodegeneration.

## Conclusion

*BRAF* V600E CNS-JXG neoplasms appear enriched in male children, associated with multifocal parenchymal CNS lesions, background CNS white matter changes, and associated *BRAF* V600E positive systemic disease manifestations in a subset, which may in turn help expand the spectrum of pediatric ECD in the post-BRAF era. A coherent multidisciplinary approach is needed for best diagnosis, including an accurate and timely pathologic diagnosis, prospective molecular investigation, and subsequent radiographic whole-body staging to evaluate disease extent, similar to adult CNS-ECD. We propose a refinement to diagnosis of CNS-JXG based on pathology, molecular, radiology, and clinical correlation with a comprehensive diagnostic algorithm that has relevance to both clinical management and treatment protocols and is also in line with the current 2016 WHO model of reporting CNS tumors [42]. An initial morphologic diagnosis would first report histology, along with any associated results from a well-validated molecular based immunostain (i.e. BRAF VE1, pERK), if available. Only after DNA-based molecular testing with sensitive testing techniques and clinical/radiographic staging are complete should an integrated final diagnosis be rendered, with description of specific sites of involvement and molecular integration. For example, in case 3 the initial morphologic diagnosis would read as: CNS-JXG, BRAF VE1 immunostain positive. Then the final integrated diagnosis may read as: Pediatric ECD (adult-type) with involvement of brain and long bones, *BRAF* V600E positive. Such an integrated final diagnosis in CNS-JXG neoplasms will allow for refinement of management with tailored treatment protocols and possible expansion of the spectrum of pediatric ECD, based on pathology, molecular and clinical/radiographic correlation in the post-BRAF era.

## Data Availability

All data generated or analyzed during this study are included in this published article and its supplementary information files.

## Abbreviations

CNS: Central nervous system
ECD: Erdheim Chester disease
ERK: Extracellular-signal-regulated kinase
JXG: Juvenile xanthogranuloma family
LCH: Langerhans cell histiocytosis
MAPK: Mitogen activated pathway kinase
RDD: Rosai -Dorfman-Destombes disease dorfman disease
